# IL-13 in LPS-Induced Inflammation Causes Bcl-2 Expression to Sustain Hyperplastic Mucous cells

**DOI:** 10.1038/s41598-017-18884-9

**Published:** 2018-01-11

**Authors:** Hitendra S. Chand, Jennifer F. Harris, Yohannes Tesfaigzi

**Affiliations:** 1 COPD Program, Lovelace Respiratory Research Institute, Albuquerque, NM 87108 USA; 2 Bioscience Division, Los Alamos National Laboratory, Los Alamos, NM 87545 USA; 3 Present Address: Department of Immunology, Herbert Wertheim College of Medicine, Florida International University, Miami, FL 33199 USA

## Abstract

Exposure to lipopolysaccharides (LPS) causes extensive neutrophilic inflammation in the airways followed by mucous cell hyperplasia (MCH) that is sustained by the anti-apoptotic protein, Bcl-2. To identify inflammatory factor(s) that are responsible for Bcl-2 expression, we established an organ culture system consisting of airway epithelial tissue from the rat nasal midseptum. The highest Muc5AC and Bcl-2 expression was observed when organ cultures were treated with brochoalveolar lavage (BAL) fluid harvested from rats 10 h post LPS instillation. Further, because BAL harvested from rats depleted of polymorphonuclear cells compared to controls showed increased Bcl-2 expression, analyses of cytokine levels in lavages identified IL-13 as an inducer of Bcl-2 expression. Ectopic IL-13 treatment of differentiated airway epithelial cells increased Bcl-2 and MUC5AC expression in the basal and apical regions of the cells, respectively. When Bcl-2 was blocked using shRNA or a small molecule inhibitor, ABT-263, mucous cell numbers were reduced due to increased apoptosis that disrupted the interaction of Bcl-2 with the pro-apoptotic protein, Bik. Furthermore, intranasal instillation of ABT-263 reduced the LPS-induced MCH in *bik* ^+/+^ but not *bik* ^−/−^ mice, suggesting that Bik mediated apoptosis in hyperplastic mucous cells. Therefore, blocking Bcl-2 function could be useful in reducing IL-13 induced mucous hypersecretion.

## Introduction

Bcl-2 is a founding member of a family of proteins that maintain cellular homeostasis by regulating apoptosis. Bcl-2 protects cells against a wide range of cell death stimuli^1,2^ by stabilizing the mitochondrial membrane and preventing permeabilization and release of death mediators^3^. Bcl-2 is inserted in the outer mitochondrial membrane and can be inactivated by binding to pro-apoptotic members of the family. While Bcl-2 is classified as an oncogene because it causes the onset of many cancers including lymphoma, it also sustains the function of thymocyte subpopulations during development^4^.

Because of its importance in various biological processes and diseases, understanding the regulation of Bcl-2 expression is very critical. Bcl-2 levels are regulated by various cytokines, including IL-1β and IGF-1 in airway epithelial cells^5,6^, IL-6 in lymphoblast cells^7^, IL-7 and IL-21 in T lymphoid cells^8,9^, IL-10 in tumor-associated macrophages^10^, andIL-22 in renal cortex tissue^11^. Many of these cytokines converge into the NF-κB pathway^12,13^ and other signaling molecules like janus kinase/signal transducer and activator of transcription (JAK/STAT)^14,15^ and phosphatidylinositol 3-kinase (PI3K)/PKB (protein kinase B)^16,17^ to increase Bcl-2 expression. However, the LPS-induced inflammatory mediators that affect Bcl-2 expression in non-hematopoietic and primary non-cancerous cells have not been extensively studied.

The airway epithelium modulates pulmonary immune responses and is a key player in the pathogenesis of chronic lung diseases^18,19^. As part of the innate immunity, airway epithelial cells (AECs) produce mucins to trap and clear inhaled particulates by mucociliary action^20,21^. In healthy subjects, few mucous cells are present in the conducting airways, but in subjects with asthma, cystic fibrosis (CF), and chronic obstructive pulmonary disease (COPD), the number of hyperplastic mucous cells increases leading to mucous hypersecretion and airway plugging^21^. MUC5AC is one of the major secretory polymeric mucins upregulated in hyperplastic mucous cells and contributes to airway reactivity^22,23^. Surprisingly, therapeutics to reduce the debilitating mucous hypersecretion are limited with only few potential drugs currently in preclinical or clinical trials^24,25^.

Following inflammatory responses to LPS or allergen exposure, Bcl-2 expression is upregulated in airway epithelial cells of animal models of mucous hypersecretion and in patients with cystic fibrosis, asthma, and chronic bronchitis^5,26,27^. Bcl-2 expression is upregulated in airway mucous cells to sustain hyperplastic mucous cells in animal models with acute and chronic inflammatory settings^26,28,29^. Therefore, the goal of the present study was to understand the pathways responsible for the coordinated induction of Bcl-2 and MUC5AC in AECs and help to identify novel intervention strategies to control mucous cell hyperplasia.

In the present study, we identified IL-13 as an important inflammatory factor that induces Bcl-2 and MUC5AC expression in response to a neutrophilic inflammation induced by LPS. When Bcl-2 function was suppressed, the role of IL-13 was switched from causing proliferation^30^ to inducing cell death in AECs in differentiated airway cultures *in vitro* and in hyperplastic mucous cells *in vivo* in a Bik-dependent manner. The small molecule BH3 domain mimetic compounds targeting the hydrophobic groove of Bcl-2 has been very successful strategy against cancer using ABT-737^31^ and it’s orally bioavailable derivative ABT-263 or navitoclax^32^. We further found that ABT-263 at very low doses alleviated LPS-induced mucous cell hyperplasia (MCH).

## Results

### LPS-induced BAL potentiates mucous cell hyperplasia and Bcl-2 expression

**Figure 1:**
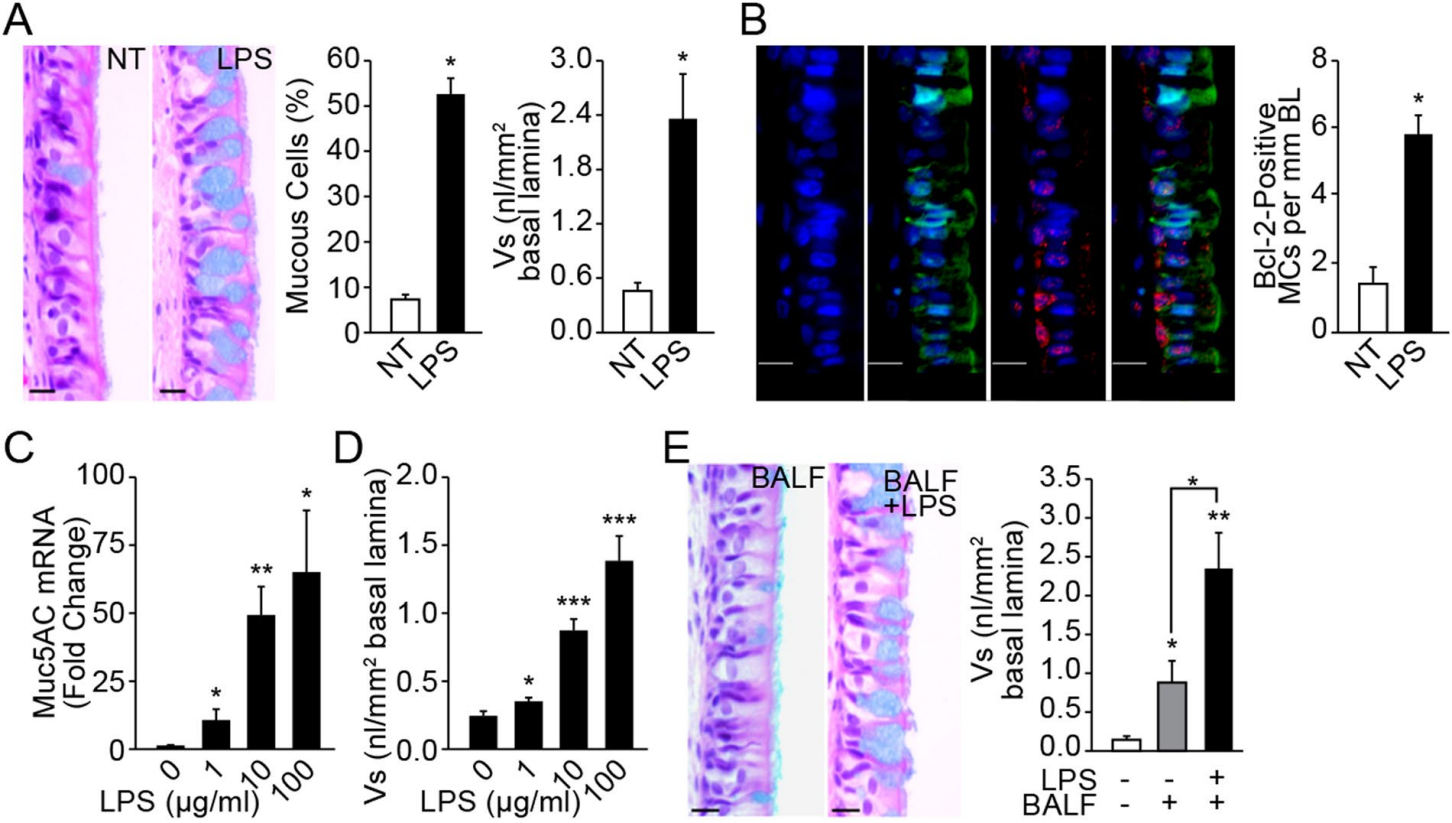
LPS exposure increases inflammatory factors in the BAL that augment Muc5AC and Bcl-2 expression. **(A)** LPS induced mucous cell metaplasia in rat nasal epithelium. Representative micrographs of nasal epithelia from non-treated (NT) and LPS-instilled rats stained with AB-PAS. Quantification of mucous cells and volume density of intraepithelial stored mucosubstances (Vs) at 3 d post LPS instillation. Data shown as mean ± SEM (n = 7/group) **(B)** LPS-induced Bcl-2 expression in mucous cells. A representative nasal epithelial section from LPS-treated rat showing Bcl-2-immunopositivity (red) among Muc5AC-positive (green) mucous cells (MCs) and the nuclei are stained with DAPI (blue). **(C)** *MUC5AC* mRNA levels in LPS-treated organ cultures quantified by q-PCR. The fold-change over non-treated controls is shown. **(D)** Quantity of the intraepithelial stored mucosubstances (Vs) in LPS-treated organ cultures stained with AB-PAS. **(E)** Representative photomicrographs of nasal explants treated with BALF from LPS-instilled rats or with BALF and 100 µg/ml LPS (BALF+LPS), and the quantity of Vs in explants at 24 h following each treatment. Data shown as mean ± SEM (n = 3/group); *p < 0.05; **p < 0.01; ***p < 0.001.

To identify inflammatory factors that induce Bcl-2 in hyperplastic mucous cells, we established a nasal epithelial explant organ culture system. We used the nasal explant culture to identify the inflammatory factors regulating Bcl-2 expression in mucous cells, because we previously have shown that nasal epithelium undergoes mucous cell hyperplasia in response to LPS injury with concomitant epithelial expression of Bcl-2^33^. The nasal explant culture avoids any alteration to the cells present *in vivo*. In addition, several studies have shown that the mucociliary epithelium of the nose has many properties that resemble the respiratory epithelium of the lung^34^. Similar to what is observed in the lung epithelium^33^, intranasal instillation of LPS caused MCH in the rat nasal distal midseptal epithelium (Fig. 1A) and the mucous cells showed increased Bcl-2 expression (Fig. 1B). Rat midseptal nasal explants when cultured on an air-liquid interface and treated with 1, 10, or 100 µg/ml LPS for 24 h and analyzed after an additional 48 h in LPS-free medium showed a dose-dependent increase in the *Muc5AC* mRNA (Fig. 1C) and in the amount of stored mucosubstances or V_s_ (Fig. 1D). However, because the quantity of stored mucosubstances was much lower than that observed *in vivo* (Fig. 1A) we postulated that inflammatory factors in the bronchoalveolar lavage (BAL) may potentiate the extent of MCH. Therefore, in addition to the 100 µg/ml LPS, explant cultures were treated with BAL fluid harvested at 24 h post LPS instillation, which results in amount of stored mucosubstances similar to that observed *in vivo* (Fig. 1E). At 24 h post LPS instillation, LPS activity in the BAL fluid was reduced drastically to 1% of the instilled amount, suggesting little contribution of the initially instilled LPS in inducing mucosubstances (Supplemental Fig. S1).

### Identification of BAL inflammatory factors that augment Bcl-2 positivity in mucous cells

**Figure 2:**
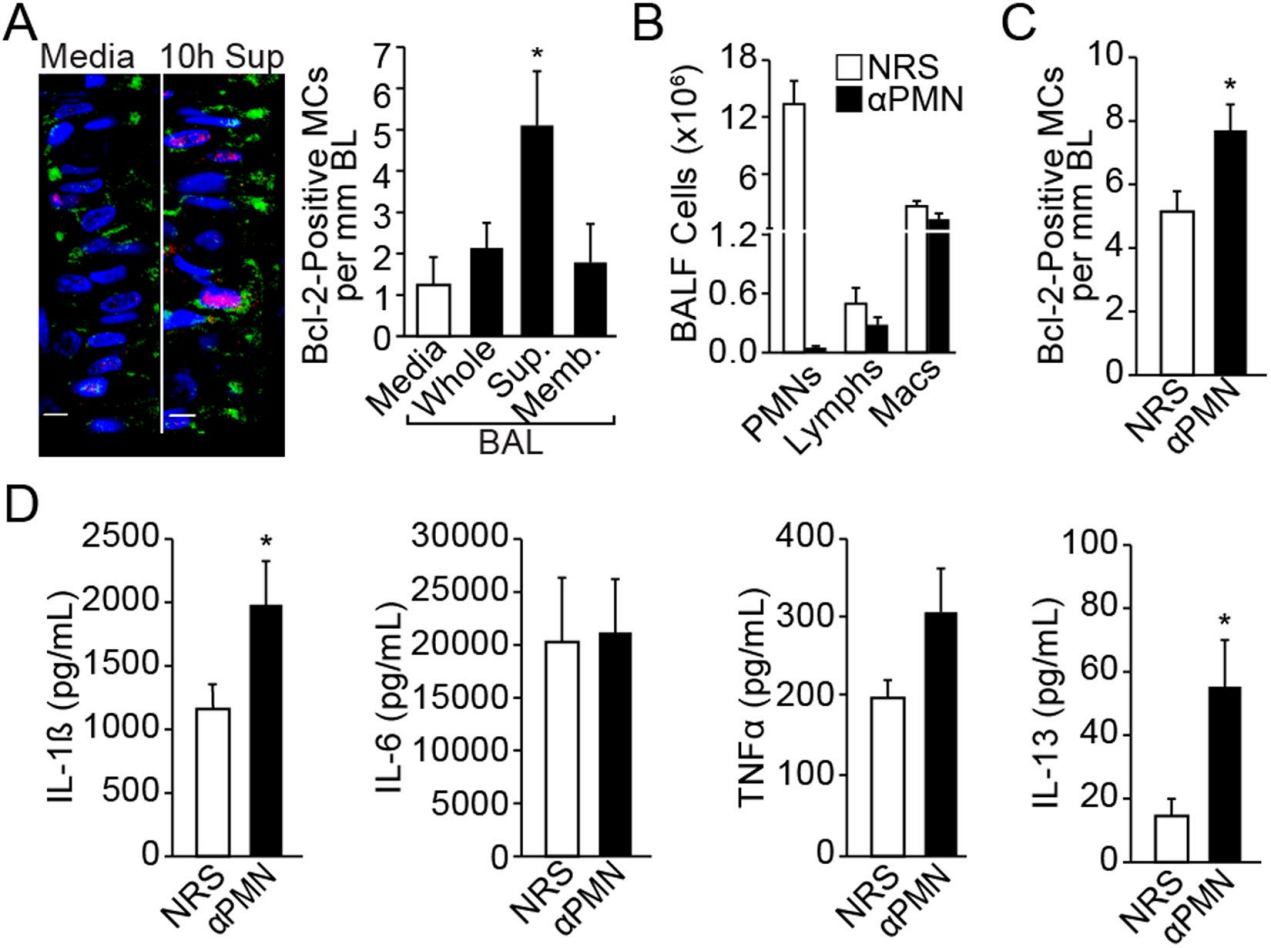
Identification of LPS-induced inflammatory factors that are required for Muc5AC and Bcl-2 expression. **(A)** Representative photomicrographs of nasal explants treated with media only or with the BAL supernatant harvested 10 h after LPS instillation showing Muc5AC- (green) and Bcl-2- (red) positivity with DAPI-stained nuclei (blue). The number of Bcl-2-positive mucous cells per mm basal lamina in the organ cultures treated with LPS-induced BAL collected at 10 h post instillation. BAL was used as Whole (BAL fluid and lavaged cells), Sup (the cell-free supernatant), or Memb (the membrane fraction prepared by lysing lavaged cells). Data shown as mean ± SEM (n = 3/group) **(B)** Rats were injected IP with anti-PMN or NRS 24 h before LPS-instillation and BAL was collected 10 h post LPS challenge. The numbers of neutrophils (PMNs), lymphocytes (Lymphs) and macrophages (Mac) in the BAL was quantified from cytospins stained with Wright-Giemsa. **(C)** Quantitation of Bcl-2-positive mucous cells in the nasal organ cultures treated with BAL supernatant from NRS- or anti-PMN treated rats collected at 10 h post LPS-instillation. Data shown as mean ± SEM (n = 5/group for NRS and n = 7/group for anti-PMN) **(D)** Inflammatory factors measured in the BAL supernatant at 10 h post LPS challenge from rats treated with NRS or anti-PMN. Data shown as mean ± SEM (n = n = 5/group for NRS and n = 7/group for anti-PMN /group); *p < 0.05.

To identify the inflammatory factor(s) responsible for induction of Bcl-2 in mucous cells, BAL was harvested at 3, 10, and 24 h post LPS instillation and fractionated into cell-free supernatant and cell membrane-bound fractions. Our previously published studies have extensively characterized the mucous cell hyperplasia and Bcl-2 expression when rats are instilled with 1000 µg/ml LPS^5,26,35^. In addition, the detailed kinetics of the inflammatory response and mucous cell hyperplasia was followed over 90 days post instillation of 1000 µg LPS and reported^36^. Therefore, we used 1000 µg LPS to identify the inflammatory cytokines that induce Bcl-2 expression. Treatment of organ cultures with the supernatant fraction of BAL harvested at 10 h post LPS instillation compared to the media-treated controls induced maximal Bcl-2-positivity and mucous cells (MCs)/mm basal lamina (BL) (Fig. 2A), while the BAL collected at 3 h post LPS instillation 15% of the LPS remaining (Supplemental Fig. S1) showed no effect (data not shown). Because we have previously observed that depletion of polymorphonuclear cells (PMNs) leads to increased Bcl-2 positivity in hyperplastic mucous cells *in vivo* ^37^, we treated the explants with BAL harvested at 10 h post LPS instillation from PMN-depleted and non-depleted controls injected with normal rabbit serum (NRS) (Fig. 2B). Consistent with Bcl-2 expression in rats depleted of PMNs^37^, explant cultures treated with BAL from PMN-depleted rats compared with controls showed increased Bcl-2 positivity (Fig. 2C). Multiplex analysis of cytokines showed increased levels of IL-1β, IL-6, TNFα and IL-13 in BAL samples from LPS- compared to saline-instilled rats, but only IL-1β and IL-13 were significantly higher in the BAL from PMN-depleted compared to NRS-injected rats (Fig. 2D). Levels of MCP-2 and GRO-KC were not affected by PMN depletion (Suppl. Fig. S2). Together, these findings suggested that IL-1β and IL-13 were the main inducers of Bcl-2 expression in hyperplastic mucous cells.

### IL-13 induces Bcl-2 and MUC5AC in human airway epithelial cells (HAECs)

**Figure 3:**
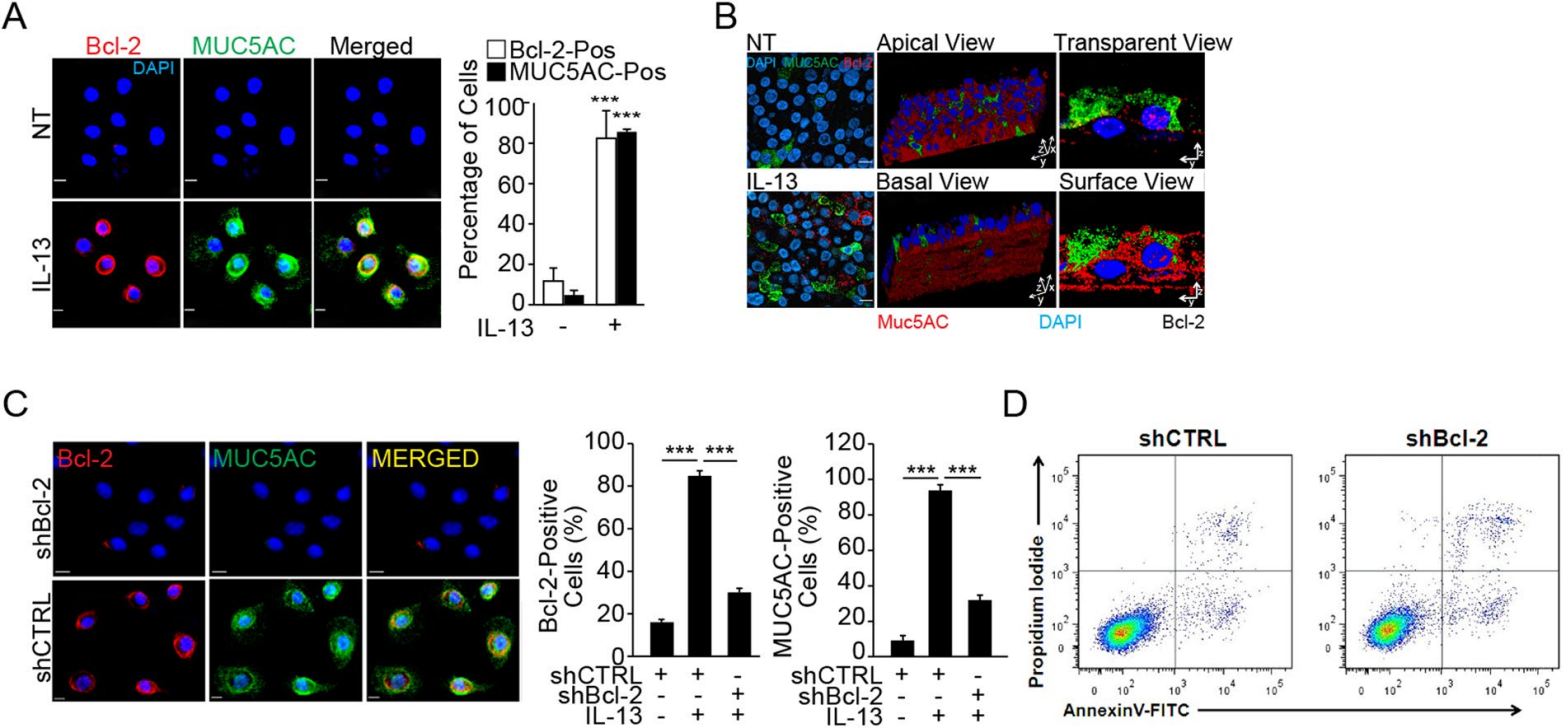
IL-13 induces MUC5AC and Bcl-2 expression in human airway epithelial cells (HAECs). **(A)** Cytometric analysis of Bcl-2 (red) and MUC5AC (green) positivity in HAECs treated with IL-13 (10 ng/ml) or media for 48 h as shown in representative micrographs in top panels with DAPI-stained nuclei (blue). Approximately 300 cells from each treatment were analyzed to calculate the percentage of Bcl-2-positive (Bcl-2-pos) and MUC5AC-positive (MUC5AC-pos) cells. Data shown as mean ± SEM (n = 10/group)***p < 0.001. **(B)** Representative micrographs of differentiated cells treated with IL-13 or left untreated (NT). Differentiated HAECs were treated with IL-13 or left untreated and were co-immunostained for Bcl-2 (red) and MUC5AC (green) and analyzed by laser-scanning confocal microscopy. A 2-D image rendering of lateral views of differentiated cells treated with IL-13 showing using transparent-rendering (left-panel) and surface-rendering (right panel) algorithms. A 3-D image rendering of differentiated cells treated with IL-13 showing apical and basal views of a rotated image of the differentiated culture mount. **(C)** Suppression of Bcl-2 expression and the effect on MUC5AC expression levels in cells transfected with shBcl-2 or shCTRL, and treated with IL-13. Representative micrographs of HAECs transfected with shBcl-2 or shCTRL, and treated with IL-13 showing Bcl-2 (red) and MUC5AC (green) immunostaining and DAPI-stained nuclei (blue). Bcl-2- and MUC5AC-positive cells were quantified. Data shown as mean ± SEM with n = 3 per treatment group. ***p < 0.001. **(D)** Analysis of apoptotic cells recovered after IL-13 treatment of untransfected (UT) or shCTRL- or shBcl-2-transfected cells. The cells were stained with Annexin V (AnnV) and propidium iodide (PI) to analyze apoptotic cells by FACS analysis. Early (AnnV) and late (AnnV+PI) apoptotic cells were increased by 4–5-fold. Data shown are representative of 3 experiments.

Our previous study had demonstrated the role of IL-1β in inducing Bcl-2 expression as discovered by microarray analyses^5^. Therefore, the present study focused on investigating the physiological importance of IL-13 in mediating Bcl-2 and mucin (MUC5AC) expression. In primary HAECs, IL-13 treatment increased the number of cells that immunostained positive for Bcl-2 and MUC5AC expression (Fig. 3A). In differentiated HAEC, Bcl-2 was localized around perinuclear and basal regions while MUC5AC was localized in the apical regions, as analyzed by 3-D imaging (Fig. 3B, middle panels) and lateral 2-D image algorithms (Fig. 3B, right panels).

### Suppression of Bcl-2 reduces MUC5AC expression by inducing apoptosis

To investigate the role of Bcl-2 in mucin expression, we blocked Bcl-2 expression using retroviral shRNA expression vector (shBcl-2). Both Bcl-2- and MUC5AC-positivity were significantly reduced in shBcl-2 compared with shCTRL cells (Fig. 3C). This reduction was due to apoptotic death of cells in shBcl-2-transfected cells at 24 h post treatment reduction as shown by Annexin V staining (Fig. 3D).

### ABT-263 treatment reduces MCH in the mouse model of LPS exposure

**Figure 4:**
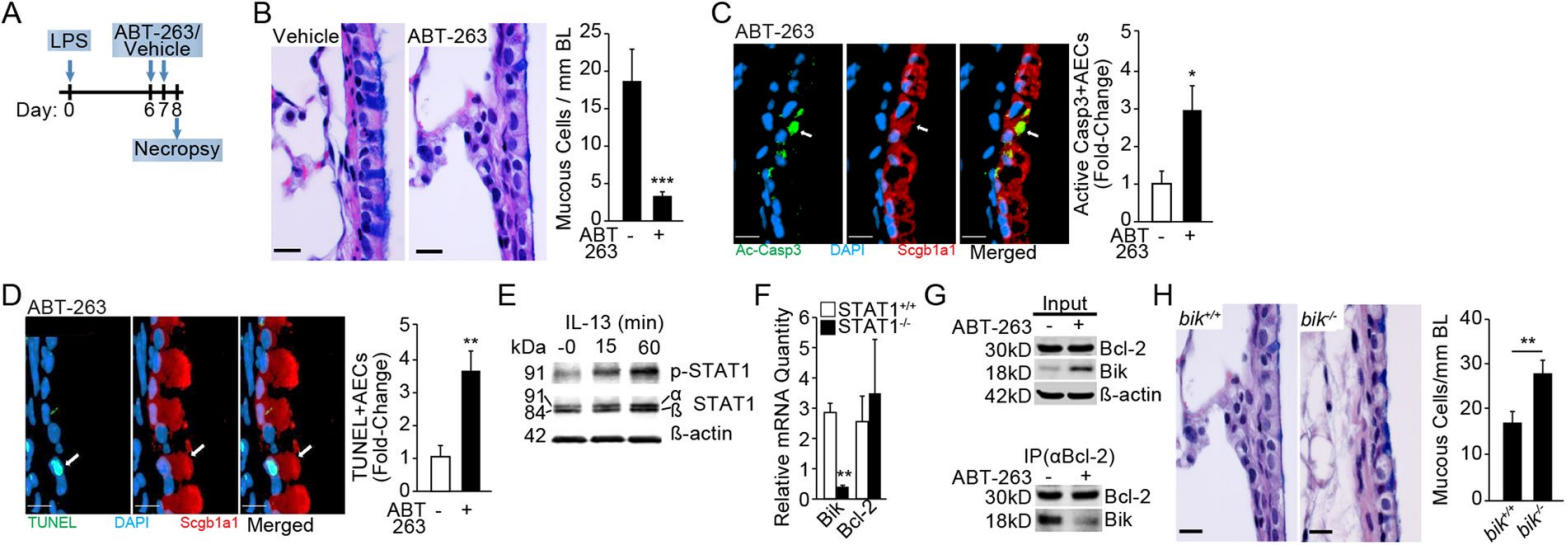
ABT-263 reduces endotoxin-induced mucous cell hyperplasia *in vivo* in a Bik-dependent manner. **(A)** Experimental outline for testing therapeutic efficacy of ABT-263 in LPS-induced MCH in mice. **(B)** Representative micrographs of lung tissue sections stained with Alcian-Blue (AB) and H&E from LPS-challenged mice treated with vehicle or ABT-263 (2 mg/Kg) are shown. Quantification of mucous cell numbers per mm BL. **(C)** Representative micrographs showing activated (cleaved) caspase 3 or Ac-Casp3 (green) among Scgb1a1-positive (red) secretory cells in mouse axial airways. The relative fold-change in the number of ac-Casp3+ secretory cells in LPS-challenged mice treated with vehicle or ABT-263. **(D)** Representative micrographs showing TUNEL-positivity (green) in Scgb1a1+ (red) secretory cells in mouse axial airways treated with ABT-263 and DAPI-stained nuclei (blue). The relative fold-change in the number of TUNEL+ secretory cells in mice challenged with LPS and treated with vehicle or ABT-263. **(E)** STAT-1 phosphorylation in HAECs following 0, 15, and 60 minutes of IL-13 treatment. Cropped Western blots are displayed. **(F)** *Bik* and *Bcl-2* mRNA levels in IL-13 treated *STAT1* ^−/−^ and *STAT1* ^+/+^ MEFs compared with the respective non-treated cells. **(G)** Immunoprecipitation with anti-Bcl-2 antibodies of proteins extracted from HAECs treated with nothing or ABT-263. Bik levels are reduced in the pull-down while increased in the flow-through of HAECs treated with ABT-263 compared with non-treated controls. Cropped Western blots are displayed. **(H)** Representative micrographs of lung tissue sections from LPS-challenged mice stained with AB-H&E and quantification of mucous cell numbers per mm BL in mice treated with vehicle or ABT-263 (2 mg/Kg) following LPS challenge. Scale = 20 µM; Data shown as mean ± SEM (n = 5–10 mice/group); *p < 0.05; **p < 0.01; ***p < 0.001.

We further investigated the therapeutic effect of blocking Bcl-2 using the small molecule BH3 mimetic, ABT-263, in the mouse model of LPS-induced inflammation and MCH^6,37^. The number of MCs/mm BL was significantly reduced in mice intranasally treated with ABT-263 (2 mg/kg body weight) for 2 consecutive days starting 6 d post instillation of mice with 50 µg of LPS (as illustrated in Fig. 4A) compared with mice instilled with vehicle (Fig. 4B). Because we failed to detect dying MUC5AC-positive mucous cells, we used Scgb1A1, a secretoglobulin detected in secretory Club cells^38^, as an alternative to identify dying secretory epithelial cells. The number of cells positive for active (cleaved) caspase 3 (Ac-Casp3) (Fig. 4C) and TUNEL (Fig. 4D) were increased among Scgb1A1-positive secretory cells in ABT-263- compared to vehicle-treated mice.

Our previous studies have established that Bik expression causes cell death in airway epithelial cells, and cells from *bik* ^−/−^ mice are resistant to IFNγ-induced cell death^39^. Therefore, we investigated whether IL-13 increases Bik expression. IL-13 activates signal transduction and transactivator 1 (STAT1) in several cell types^40^, a transcription factor responsible for Bik induction^39^. We found that IL-13 in HAECs phosphorylated STAT1 at 15 and 60 min of treatment (Fig. 4E) and thereby increased Bik expression, because *STAT1* ^−/−^ compared with *STAT1* ^+/+^ mouse embryonic fibroblasts (MEFs) when treated with murine IL-13 showed significantly lower *Bik* mRNA levels, while Bcl-2 levels remained unaffected (Fig. 4F). In addition, the amount of Bik protein that immunoprecipitated by Bcl-2 antibodies was significantly reduced by ABT-263 and remained in the input of ABT-263-treated cell extracts (Fig. 4G), suggesting that ABT-263 disrupted Bik/Bcl-2 interaction. The importance of Bik in the resolution of LPS-induced MCH was assessed by exposing *bik* ^+/+^ and *bik* ^−/−^ mice to ABT-263 or vehicle following LPS challenge. ABT-263 treatment suppressed the MCH in *bik* ^+/+^ but not in *bik* ^−/−^ mice (Fig. 4H) confirming the role of Bik in causing cell death in hyperplastic mucous cells when Bcl-2 is blocked with ABT-263.

## Discussion

Our previous studies showed that exposure of rodent lungs to LPS causes extensive neutrophilic inflammation and mucous cell hyperplasia that is sustained by Bcl-2 expression in epithelial mucous cells^26,27,33,35,36,41^. By using BAL fluid from rats depleted of PMNs, the current studies identify IL-13 as the factor that induce MUC5AC and Bcl-2 expression in airway cells. Because Bcl-2 sustains hyperplastic mucous cells, targeted inactivation of Bcl-2 using the BH3 mimetic, ABT-263, suppresses LPS-induced mucous cell hyperplasia in a Bik-dependent cell death pathway.

LPS-induced inflammation is characterized by an initial influx of large number of PMNs over the first 3–8 h followed by macrophages and lymphocytes over 6–12 h post LPS instillation^36^. The inflammatory factors derived from these cells affect the airway epithelium to establish mucous cell hyperplasia over 48–72 h post instillation^37,41,42^. We were able to replicate the extent of MCH observed in the nasal midseptum *in vivo*, when treating the organ culture system with BAL fluid harvested from 10 h post LPS instillation. However, the whole BAL that included lavaged cells did not induce Bcl-2 expression, suggesting that the inflammatory cells released contents that inhibit Bcl-2 expression. The effect of additional LPS from the BAL fluid is minimal, given that we had already added 100 μg/ml LPS to the explant culture. If higher LPS levels was causing Bcl-2 positivity, BAL fluid collected at 3 h post LPS-instillation that had even higher levels of LPS would be expected to induce Bcl-2 expression, but showed no significant change in Bcl-2 expression. These findings suggest that the Bcl-2-inducing IL-13 and IL-1β reach maximum levels at 10 h post instillation, because BAL fluid harvest at 3 or 24 h was not effective in inducing Bcl-2 expression. Significant neutrophilic inflammation sets in within 3 hour of LPS exposure in rodents as well as in humans^36,43^. However, the effect of neutrophils on other cell types is highly complex, given that the lung consists of many cell types. Our previous study demonstrated that depletion of PMNs caused an increase in Bcl-2-positive mucous cells *in vivo* ^37^. The *ex vivo* midseptum culture also showed increased Bcl-2 positivity when treated with BAL from rats depleted of PMNs, suggesting that the organ culture system reliably replicated the *in vivo* findings. Analyses of cytokines that are differentially increased in BAL fluid from PMN-depleted compared to non-depleted controls allowed us to identify IL-13 as one of the inflammatory factors responsible for Bcl-2 expression in mucous cells. While LPS-induced inflammation is primarily known to increase the cytokines IL-1β, IL-6, TNF-α^37^ and IL-18^36^, macrophages from various rat strains, when stimulated with anti-CD8 antibody *in vitro*, also produce IL-13^44^. Many studies have shown that IL-13 is expressed by several inflammatory cells including T helper 2 (T_H_2) cells, type 2 innate lymphoid cells (ILC2s), invariant natural-killer T (iNKT) cells, eosinophils, or alternatively activated macrophages^45^. However, while T cells are found in the BAL of LPS-instilled rats^36^, these T cells do not produce T_H_2 cytokines. Rather other cell types, including endothelial cells and epithelial cells produce IL-13 when rats are challenged with LPS^30,46^. Further, LPS at low concentrations induces IL-13 production from mast cells by activating TLR2 and TLR4 receptors^47–49^. Therefore, depletion of neutrophils may have enriched for the IL-13 detected in the BAL of LPS-instilled rats and facilitated increased expression of Bcl-2 in hyperplastic mucous cells.

IL-13 plays an important role in proliferation and repair of airway epithelial cells and promotes mucous cell hyperplasia^50–52^ by inducing expression of the Sam pointed domain-containing ETS transcription factor (SPDEF) in Club cells through a STAT6-dependent mechanism. Because Bcl-2 and MUC5AC expression have repeatedly been observed to occur within the same cells^26,29,33^, it is likely that IL-13 may activate the same pathways to induce expression of these two genes. Similar to MUC5AC^53^, induction of Bcl-2 expression is mediated through transactivation of EGFR pathway^5^, and EGFR activation is necessary for IL-13–mediated MCH^30,54,55^. IL-13 indirectly activates EGFR via production of TGF-α^56^, HB-EGF^30^, or epigen^57^. IL-13–induced TACE and release of TGF-α is also directly implicated in the airway epithelial hyperproliferation^56^. Therefore, airway IL-13 levels may be critical for normal cellular homeostasis in the setting of airway epithelial injury because it coordinates the proliferative and cytoprotective activity. Similarly, IL-13 when co-incubated with IL-9 is protective against spontaneous or corticosteroid-induced apoptosis by upregulating Bcl-2^51,58^. Unfortunately, therapeutic targeting of IL-13 results in adverse events because this cytokine also elicits immunoregulatory functions^59^. For instance, IL-13 suppresses Th17 cytokine production in an IL-10-dependent manner^60^ and thereby may play an important role in Th17-associated autoimmune diseases like multiple sclerosis, rheumatoid arthritis, and colitis^61^. Consequently, IL-13–targeted therapeutics result in significant adverse events involving the musculoskeletal diseases that are associated with Th17 cytokines^62^. Therefore, targeting of Bcl-2 function to reduce MCH by a Bik-mediated cell death is an approach that is more specific to mucous cells and likely to have less side effects.

Bcl-2 has been found in the nucleus, as well as associated with the ER^63^ or mitochondria^64^. However, in differentiated HAECs of Bcl-2 was localized in the basal and peri-nculear areas while MUC5AC was enriched in the epical region as previously reported^65,66^. Although we have reported that suppression of Bcl-2 causes reduction of mucous cells^26^, the inducer of cell death when the anti-apoptotic Bcl-2 was downregulated, had not been identified. The present study demonstrates that IL-13 increased expression of Bcl-2 and Bik to alter the airway epithelial cell fate and that the pro-apoptotic Bik is required for ABT-263 mediated suppression of LPS-induced MCH. Our previous studies have established that Bik expression causes cell death in airway epithelial cells as *bik* ^+/+^ but not *bik* ^−/−^ mice resolve MCH during prolonged exposure to allergen, and *bik* ^*−/−*^ cells are resistant to IFNγ-induced cell death^39^. In addition, clinical findings show that *Bik* mRNA levels are significantly reduced in airway cells of asthmatics^39^ and chronic bronchitics^67^ compared to non-diseased controls. Most importantly, Bik expression causes cell death only in proliferating airway epithelial cells^67^ but not in other cell types, such as hematopoietic and endothelial cells^68^. ABT-263 has been successfully used in Phase II clinical trials for cancer treatment^32,69^. The dose used for cancer patients is 250 mg/d over 21 days. In contrast, we found efficacy in reducing LPS-induced MCH at doses that are a 100-fold lower. Therefore, the small molecule Bcl-2 inhibitors, when delivered directly to the lung, may provide better treatment options against mucous hypersecretion.

The utility of rat nasal organ culture to identify the regulatory mechanisms underlying MCH and Bcl-2 expression in the lower airways supports the recent findings that cells of the upper airways strongly resemble the airway cells lining the lung airways^70,34^. The upper and lower airway diseases may display different manifestations of the same inflammatory process^71^. Therefore, suppression of Bcl-2 expression or blocking Bcl-2 function as shown in this study may also have beneficial effects in rhinosinusitis in the context of chronic mucus hypersecretion.

The present studies provide a novel paradigm to primarily target hyperplastic mucous cells by suppressing Bcl-2 function and thereby switching the proliferative function of IL-13 into an efficient suppressor of MCH. These findings support the development and use of the small molecule Bcl-2 inhibitors as a novel treatment modality for patients with cystic fibrosis and chronic bronchitis when delivered directly to the lung. ABT-263 is currently being tested for cancer therapy, specifically in lymphomas and leukemia^32^, and for senescent stem cells^72^. Together with the previous findings in a mouse model of asthma^73^, the findings suggest that this compound may also represent an effective treatment for targeting hyperplastic mucous cells. Whether blocking Bcl-2 may have therapeutic effects in chronic diseases other than LPS- and allergen-induced mucus hypersecretion that are mediated by IL-13 needs to be explored in the future.

## Methods

### Laboratory Animals

Specific pathogen-free F344/NCrR male rats, 6–8 wk of age, were obtained from NCI (Frederick, MD) and were housed until 8–10 weeks of age. Rats were housed in pairs and were provided food and water *ad libitum*, a 12-h light/dark cycle at 22.2 °C, and 30–40% humidity. The *bik* ^−/−^ mice on C57BL/6 were made available by Dr. Andreas Strasser (Walter and Eliza Hall Institute) and bred at the Lovelace Respiratory Research Institute (LRRI) and genotyped as described^68^. Pathogen-free wild-type C57BL/6J mice were purchased from The Jackson Laboratory or from in-house breeding. Rodents were housed in isolated cages under specific pathogen-free conditions. All experiments were approved by the LRRI Institutional Animal Care and Use Committee and were performed in accordance with relevant guidelines and regulations at LRRI. LRRI is a facility approved by the Association for the Assessment and Accreditation for Laboratory Animal Care International.

### LPS Challenge

F344/NCrR male rats (NCI, Frederick, MD) of 6–8 wk of age were briefly anesthetized with 5% isoflurane in oxygen and instilled intratracheally with 1000 μg of LPS (*Pseudomonas aeruginosa* serotype 10, Sigma, St. Louis, MO) in 0.5 ml of 0.9% pyrogen-free saline. Control rats were instilled with 0.5 ml of 0.9% pyrogen-free saline. Rats were sacrificed at 72 h post LPS-instillation for lung tissue analysis and BAL was collected at various time-points post LPS-instillation as described below. Similarly, C57BL/6 mice (both male and female) at 6–8 wks of age were briefly anesthetized with 5% isoflurane in oxygen and instilled intranasally with 50 μg of LPS (*P. aeruginosa* serotype 10, lot 31K4122, 3,000,000 LPS units (EU)/mg, Sigma, St. Louis, MO) in 0.05 ml of 0.9% pyrogen-free saline. One group of mice received ABT-263 (0.05 mg/Kg in 0.05 ml of 0.9% pyrogen-free saline) intranasally on day 5 and 6 after LPS challenge. Control mice were instilled with 0.05 ml of 0.9% pyrogen-free saline. Mice were sacrificed 24 h post last exposure and lung tissues were processed and analyzed.

### Preparation and treatment of rat nasal epithelial organ cultures

The preparation and LPS treatment of explant cultures from rat nasal midsepta was essentially as described previously^74^. Briefly, following exsanguination, after removing the lower jaw the head was split in half longitudinally and the nasal midseptum and maxilloturbinates removed by microdissection using a Leica MZ 7.5 stereo zoom microscope (Leica Microsystems, Inc., Bannockburn, IL). The septum was cut into three sections, one proximal and two distal. The explants were cultured for 72 h by placing them epithelium side-up in transwell dishes (Corning, Incorporated Life Sciences., Acton, MA), and cultured in supplemented Ham’s/F-12 media (HyClone, Logan, UT). The nasal explants were treated with LPS for 24 h and replenished with fresh media for maintaining cultures over 48 h. We selected this time-point based on our previous studies showing that Bcl-2 expressing hyperplastic mucous cells by LPS *in vivo* peaks at 48 h post instillation^26^. Following treatments, explant cultures were fixed in zinc formalin for a minimum of 24 h, embedded in paraffin, and processed for microscopy.

### Neutrophil depletion and bronchoalveolar lavage fractionation

Rats were intraperitoneally (i.p.) injected with 1 ml of rabbit anti-rat polymorphonuclear neutrophil (PMN) antiserum or with normal rabbit serum (NRS) as control (#AIAD11540, Accurate Chemical Corp., Westbury, NY) 24 h before LPS-instillation. Rats were sacrificed at 3, 10, and 24 h post-instillation with an injection of pentobarbital sodium and exsanguinated through the renal artery. Lungs were removed with cannulated trachea, placed in ice-cold saline for 3 min, and then lavaged three times with 5 ml of Ham’s F-12 media. BAL for treatment of organ cultures was used immediately after collection and fractionation, or was stored in 0.2-ml aliquots at −80 °C until further use. For fractionation, BAL was centrifuged at 1000 × g for 10 min at 4 °C, the supernatant was separated from the cell pellets and kept at −80 °C until needed. To isolate the cell membrane fraction, cells were lysed in cold (4 °C) sterile milli-Q-filtered water, centrifuged, and the supernatant from the lysed cells was discarded. The lysis was repeated twice to achieve complete lysis and culture medium was added to the membrane fraction to obtain the original volume of the BAL.

### LPS quantification

The amount of LPS recovered in the BAL fluid at 3, 10, and 24 h post instillation was assayed using the Cambrex LAL Limulus Amoebocyte Assay (Walkersville, MD) according to package directions. Values are expressed as percentage of 1000 μg initially instilled intratracheally.

### Histological Analysis

Histochemical staining for Alcian Blue and periodic acid Schiff (AB-PAS) was carried out as previously described^35^. Airway epithelial cell and mucous cell numbers per mm basal lamina (BL) were measured by counting the number of nuclei and mucous cells, respectively, and dividing by the length of the BL. Images were taken using a light microscope (Eclipse E600W; Nikon) with a Plan Fluor 60× NA 0.85 objective and a digital camera (DXM1200F; Nikon) with ACT-1 acquisition software (version 2.62 l Nikon). In all cases, the VisioMorph system (Visiopham A/S, Horsholm, Denmark) was used for morphometry by a person unaware of slide identity.

### Luminex Analysis

The levels of cytokines and chemokines (IL-1β, IL-6, IL-13, TNFα, Gro/KC, and MCP-1) in BAL fluid were determined using a multiplex assay kit (Lincoplex panel, Linco Research, Inc., St. Charles, MO) according to the manufacturer’s instructions. Briefly, the BALF was filtered to remove cells and debris, then beads were incubated with diluted standards, or BALF overnight followed by a detector antibody cocktail for 60 min each at room temperature. After two washes in PBS supplemented with 0.02% Tween 20, 0.1% BSA, and 0.02% NaN3, the beads were incubated for 30 min with fluorescent dye-conjugated streptavidin. Cytokine levels were measured using a flow cytometer and were analyzed with Flowmetrix software (Luminex, Ausitn, TX). Standard curves for each cytokine and chemokine were generated on a log-log plot for each assay, and the concentrations in each sample were calculated from the corresponding curve-fitting equations.

### Immunofluorescence analysis

Tissue sections were deparaffinized, hydrated in graded ethanol and deionized water, then washed in 0.05% v Brij-35 in Dulbecco’s PBS (pH 7.4). The antigens were unmasked by treating with Digest-All kit (Zymed Laboratories, San Francisco, CA) at a 1:3 dilution of trypsin to diluent at 37 °C for 10 min. Sections were then blocked using 0.2% Triton X-100 with 0.2% Saponin in a blocking solution containing 3% IgG-free BSA, 1% Gelatin and 2% normal donkey serum followed by incubation with anti-Bcl-2 (#sc-492, Santa Cruz Biotech, CA), anti-MUC5AC (#MAB2011, Millipore Inc.), anti-active Caspase 3 (#9661, Cell Signaling Technologies, CA) or isotype controls (Cell Signaling Technologies, CA). The immunolabeled cells were detected using F(ab)_2_-fragments of respective secondary antibodies conjugated to either Dylight^TM^-549 or Dylight^TM^-649 (Jackson Immunoresearch, West Grove, PA) and mounted with 4′,6-diamidino-2-phenylindole (DAPI) containing Fluormount-G^TM^ (SouthernBiotech, Birmingham, AL) for nuclear staining.

For cytometry, cells were grown on Lab-Tek-II 8-chamber slides (Nalgene Nunc International, Rochester, NY) and treated with 10 ng/ml of IL-13 or were left untreated and were fixed using 3% paraformaldehyde with 3% sucrose in PBS and processed for immunostaining as described above. Micrographs were captured using either Zeiss LSM 510 Meta confocal microscope (Carl Zeiss MicroImaging, Inc, Thornwood, NY) or using the Axioplan 2 fluorescent imaging system (Carl Zeiss, Thornwood, NY) equipped with a charge-coupled device camera (ORCA-ER; Hamamatsu Photonics, Iwata City, Japan) and SlideBook 6 acquisition software (Intelligent Imaging Innovations, Denver, CO). Quantification of Bcl-2-positive and MUC5AC-positve cells per mm of basal lamina was performed using the VisioMorph system (Visiopham A/S, Horsholm, Denmark) or NIH ImageJ (http://imagej.nih.gov/ij/) software. Cells cultured in air-liquid interface were also immunostained for Bcl-2 and MUC5AC similarly.

### TUNEL Assay

For detection of apoptotic cells, deparaffinized lung sections were stained using TACS® 2 TdT Fluorescein Kit (Trevigen Inc., Gaithersburg, MD) and fluorescent TUNEL-positive cells were detected as described earlier for fluorescent staining. In all cases, quantification of TUNEL-positivity was carried out by a person unaware of slide identity.

### Cell culture

The human airway epithelial cells (HAECs) were maintained in bronchial epithelial growth medium (BEGM, Lonza, Walkersville, MD). Primary HAECs were purchased from Clontech (Walkersville, HD). For air-liquid interface culture primary HAECs were seeded on Transwell membranes and differentiated for 14 days. Following treatments, the membrane quarters were used for qRT-PCR and membrane halves embedded in paraffin for immunostaining. Cell viability was determined by trypan blue exclusion.

### Immunoprecipitation and Western blot analysis

Protein was extracted from cells or tissues by homogenization in RIPA buffer (10 mM Tris, pH 7.4, 150 mM NaCl, 1% Triton X-100, 1% deoxycholate, 0.1% SDS, and 5 mM EDTA) supplemented with a protease inhibitor cocktail (Sigma Chemical Co., St. Louis, MO). Protein concentration was determined using the BCA kit (Pierce, Thermo Fisher Scientific, Rockford, IL) and 50–100 μg of protein lysate was analyzed by Western blotting. For immunoprecipitation using the Pierce Crosslink IP Kit (# 26147, Thermo Fisher Scientific), cells were rinsed twice with cold PBS, scraped into cold PBS plus protease inhibitors and analyzed per manufacturer’s instructions. The Bcl-2 (#sc-7382) antibody was from Santa Cruz Biotechnology Inc., CA) and antibodies to Bik (#4592), p-STAT1 (#9167) and STAT1 (#9172) were from Cell Signaling Technologies (Boston, MA). The proteins were detected using appropriate peroxidase-conjugated secondary antibodies and visualized by chemiluminescence (Perkin Elmer, Waltham, MA) using the FujiFilm Image Reader LAS-4000 (Valhalla, NY).

### Quantitative RT-PCR

RNA was isolated from the snap-frozen right lungs of animals using TRIzol as described previously^39^ whereas RNA from cultured cells was extracted using the RNeasy kit (Qiagen, Valencia, CA) and concentration was determined using the Thermo Scientific Nanodrop 1000 Spectrophotometer (Thermo Fisher Scientific, Waltham, MA). The primer/probe sets for *MUC5AC* and *CDKN1B* were obtained from Life Technologies (Carlsbad, CA) and were amplified by quantitative real-time PCR using RT-PCR Master Mix (Life Technologies (Carlsbad, CA)) in the ABIPRISM 7900HT Real-Time PCR System. Relative quantities were calculated by normalizing averaged C_T_ values to *CDKN1B* to obtain ΔCt, and the relative standard curve method was used for determining the fold change as described previously^75^.

### Blocking Bcl-2 with retroviral transfection and ABT-263

HAECs were transfected with Bcl-2 shRNA containing retroviral vectors or control vectors (Origene Technologies, Inc., Rockville, MD) as per manufacturer’s instructions. After infection with Bcl-2 or control shRNAs, cells were treated with 10 ng/ml human IL-13, and 48 h later cells were assessed for Bcl-2 expression by immunofluorescence or Western blotting.

### Statistical analysis

Grouped results were expressed as means ± SEM. Data were analyzed using GraphPad Prism Software (GraphPad Software, Inc., San Diego, CA). Grouped results were analyzed using analysis of variance. We performed the Kruskal–Wallis 2-sample non-parametric H test to compare the NRS and anti-PMN groups for the sample comparisons of unequal sample size. When significant main effects were detected (*P* < 0.05), Fishers least significant difference test was used to determine differences between groups. In addition, data were log transformed to correct for possible heteroscedasticity and reanalyzed for statistical differences. For all analyses the results remained unchanged.

## Electronic supplementary material

Supplementary Information
