# Supplementary material for: IL-13 in LPS-Induced Inflammation Causes Bcl-2 Expression to Sustain Hyperplastic Mucous cells
Source: Sci Rep. 2018 Jan 11;8:436. doi: 10.1038/s41598-017-18884-9 (PMC5765145; doi:10.1038/s41598-017-18884-9)
Supplement: Supplementary file 1 — Supplementary Information [file 41598_2017_18884_MOESM1_ESM.doc]

**Supplemental Material**

**IL-13 in LPS-Induced Inflammation Causes Bcl-2 Expression to Sustain Hyperplastic Mucous cells**

Hitendra S. Chand ^1,2^ , ^1,3^Jennifer F. Harris, and Yohannes Tesfaigzi ^1^,*****

^1^COPD Program, Lovelace Respiratory Research Institute, Albuquerque, NM 87108, USA, ^2^ Present Address: Department of Immunology, Herbert Wertheim College of Medicine, Florida International University, Miami, FL 33199; ^3^Bioscience Division, Los Alamos National Laboratory, Los Alamos, NM 87545;


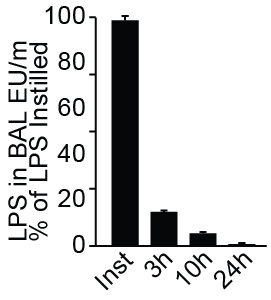


**Figure S1: LPS in the BAL is rapidly reduced over time.** Rats were instilled with 1000 μg LPS and the amount of LPS recovered in the broncho-alveolar lavage at 3, 10, and 24 h was measured using the Limulus Amoebocyte Assay and expressed as percentage from that instilled. Data shown as mean±SEM (n = 3 rats/group).

**
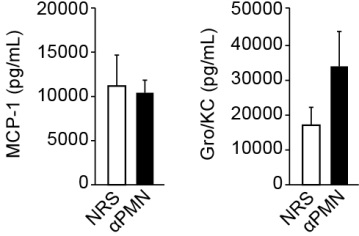
**

**Figure S2. LPS-induced BAL inflammatory factors in PMN-depleted rats.** There were no significant differences in the levels of inflammatory chemokines, MCP-1 and GRO/KC in the BAL supernatant at 10 h post LPS challenge from rats treated with NRS or anti-PMN. Data shown as mean±SEM (n=5-10/group).
